# Anaerobic co-digestion of khat waste and cow dung for enhanced bio-methane production potential: Substrates conversion rate

**DOI:** 10.1016/j.heliyon.2024.e41124

**Published:** 2024-12-11

**Authors:** Henok Akililu Legesse, Wagene Hailu Debele, Akiber Chufo Wachemo

**Affiliations:** a Department of Water Supply and Environmental Engineering, Arba Minch Water Technology Institute (AWTI), P. O. Box 21, Arba Minch, Ethiopia; b Adigrat University, College of Engineering and Technology (CET), Department of Civil Engineering, Water Supply and Environmental Engineering Stream, P. O. Box 50, Adigrat, Ethiopia; c Departments of Water Supply and Environmental Engineering, Arba Minch Water Technology Institute (AWTI), P. O. Box 21, Arba Minch, Ethiopia

**Keywords:** Anaerobic Co-Digestion, Cow dung, Biomethane, Khat waste, Substrate conversion

## Abstract

Anaerobic digestion technology is one of the most paramount eco-friendly wastes to energy conversion processes. This study was conducted to characterize the physicochemical properties of khat and Cow dung along with examining the bio-methane production potential and substrate conversion rate of feedstock through seven triplicate proportions of laboratory scale batch anaerobic reactors for a 27 days digestion period under mesophilic conditions. The maximum and minimum bio-methane yield of 283.52 ± 7.17 CH_4_ mL/g VS and 142.83 ± 3.56 CH_4_ mL/g VS were generated from the digester, with the higher proportion of Khat waste in the T-5 (2:1) and the sole substrate anaerobic digestion of Cow dung in T-7 (0:1) respectively. The conversion rates of cellulose and hemicellulose components from Khat waste were 44.4 and 47.2 %, respectively. The result demonstrates that the anaerobic co-digestion of khat waste and cow dung plays a critical role in enhanced biomethane production due to effective synergism.

## 1 Introduction

Globally solid waste generation stands at around 1.3 billion tonnes per year and is predicted to rise to 2.2 billion tonnes per year by 2025. Moreover, in the next fifteen years per capita solid waste generation rates will rise significantly from 1.2 to 1.42 kg per person per day. Particularly, in sub-Saharan Africa, waste generation rates are estimated to be 62 million tonnes per year with an average of 0.65 kg per capita per day [1]. Correspondingly, in urban and rural areas of Ethiopia, solid waste generation varies from 0.17 to 0.48 and 0.11–0.35 kg per capita per day respectively [2].

Biodegradable waste takes the largest fraction of municipal solid waste. Particularly, most solid wastes in developing countries are fermentable in nature [2]. However, improper solid waste disposal has significant environmental consequences in terms of soil, water, and air quality around the world [3]. However, proper management of solid waste leads to economic benefits and reduces environmental pollution [4].

Anaerobic digestion (AD) is the process of breaking down an organic substrate using microorganisms to provide biogas in the absence of oxygen by bacterial flora [5]. Hence, biogas is a foul-smelling gas that contains 50–70 percent methane, 30–50 percent carbon dioxide, and various trace gases [6].

Upgrading of biogas has gained an increased target for renewable fuel production in many countries. Particularly, in Ethiopia most of the rural and poor urban communities largely depend on the use of biomass consisting of firewood, charcoal, twigs, straw, crop residue, and animal dung as a source of fuel [7]. For this reason, the Ethiopian government has attempted to reduce dependence on biomass as a source of energy to improve environmental conservation, human health, and poverty reduction in rural and poor urban households [8].

In many countries, the conversion of organic solid waste to biogas has become increasingly popular in recent years as a sustainable technology product in the form of green energy [9]. In Ethiopia, there are different organic wastes produced annually by different activities like agriculture, industries, municipal, and agro-industries. Agricultural wastes are the most significant materials used for biogas production [10].

The three types of polymers that make up lignocellulose components are cellulose, hemicellulose, and lignin. Lignocellulosic waste is a kind of waste derived from forestry, agriculture, and agro-industrial operations that accumulates in huge amounts daily [11]. Physical, chemical, physicochemical, enzymatic, and microbiologic techniques of biomass pretreatment are used to prepare lignocellulosic materials as substrates for biomethane production [12]. Chemical pretreatment is one of the viable pretreatment techniques because it is more successful in enhancing the biodegradation of complex compounds and the anaerobic digestion performance of agricultural wastes [13].

Khat (Catha edulis Forsk) is a stimulant and an evergreen tree that is cultivated for its fresh leaves, which are chewed for recreational purposes [14]. According to the [15] report the total area cultivated in the year 1998 was estimated at 78,570 ha, in 2008 increased to 163,227 ha, and in 2017 it reached 255,401 ha.

Khat is one of the most exported crops after coffee and oilseeds [10]. For instance, the industry constitutes 4 % of the country's export earnings and shares 9.4 % of total merchandise exports [16]. Furthermore, the value received from exported Khat increased from 15.9 million ETB in 1985 to 618.8 million ETB in 2000 and continued to rise and reached 6.1 billion ETB in 2017 [17].

Export packagers and chewers discard older leaves and twigs, generating a huge amount of solid trash. Every day, khat trash accumulates in the city, polluting the environment [10]. Specifically, the rate of solid waste generation in Dire Dawa City has increased along with the rate of population expansion, and the City municipal is capable of collecting 48 % of the remaining 52 % discharged as solid garbage in the drainage lines, open space, and roadway sides [18].

Improper management of khat waste creates health problems, releases a bad odor that creates diseases, and pollutes the environment. Besides, the increasing number of people using khat, its disposal in the ditches, opens space and drainage system problems in the urban area and blocks the drainage system which is detrimental to the environment and the health of the population. The Cow dung burnt in the kitchen was used for cooking purposes. Burning dried cow dung pollutes the air, hurts people's respiratory tracts, and causes diseases including persistent cough and chronic bronchitis.

Therefore, the main objective of this study was to examine the physiochemical properties of Khat waste and Cow dung as a potential feedstock for biomethane production and determine the rate of substrate conversion to biomethane component after the AD process using batch anaerobic reactors for 27 days of digestion period. Consequently, the result of the study could have a significant contribution to the efforts of maximizing renewable energy production for desired use in urban areas and enhancing the efficient use of waste for green technology.

## 2 Materials and methods

### 2.1 Description of the study area

**Fig. 1:**
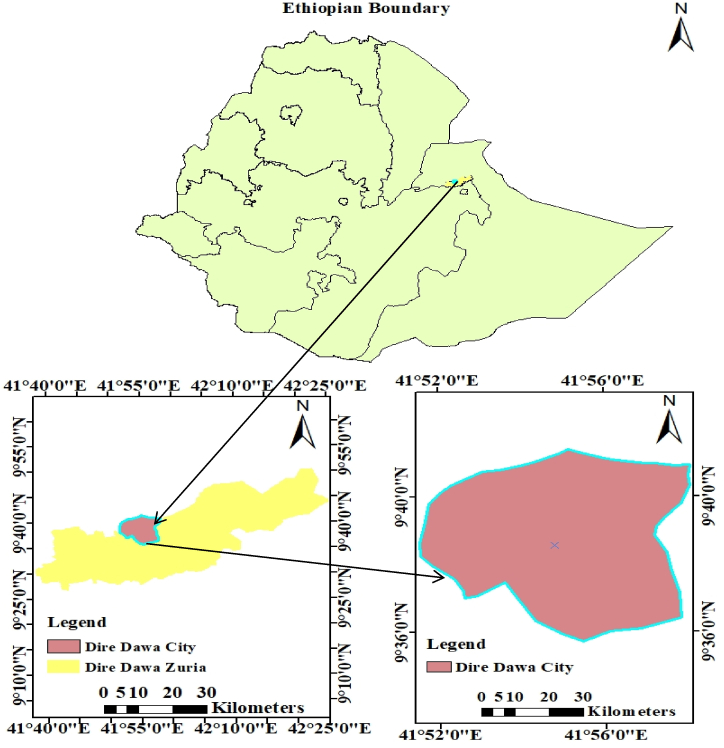
Map of the study area of Dire Dawa city administration.

Dire Dawa city administration was founded in eastern Ethiopia; it is the second-largest city with over 440,000 inhabitants. Geographically it is located between 9°27′N to 9 54′N latitude and 41°38′E to 42°19′E longitude. The city is about 515 km far away from Addis Ababa and 311 km to the west of Djibouti port, with an elevation range of 1276 m to 1285m above m.s.l. The city is located in the East African rift valley, with an average temperature and rainfall range of 21.5–32.8 °C and 568–637 mm respectively. Furthermore, the map showing the study area of Dire Dawa city administration is depicted in Fig. 1.

### 2.2 Data collection

Samples of the khat wastes were collected from Dire Dawa city administration, Ethiopia. The primary sources of data regarding Khat waste generation were acquired through a field survey, and Table 1 shows the name of kebeles, the percentage of stem and leaf of KW as well as a sample of typical khat waste from 10 distinct kebeles. A sample of khat waste was taken in a different area of the city from, randomly selected clusters of 10 kebeles of khat chewing houses; garages, streets, and shops. Therefore, this approach may help to estimate the amount of khat waste and the net proportions of the stems, and leaves of khat that are discarded as waste in the city.

**Table 1:** Table showing the name of 10 kebeles along with the average weight of khat waste collected with the percentage of stems and leaves in KW.

Sample Location	Average khat weight in g for 10 days (before chewing)	Average khat weight in g for 10 days (after chewing)	Average khat waste for 10 days after chewing (%)	KW % of stem	KW % of leave
Gendekore	486	370	76.13	73.07	26.92
Megala	630	392	62.22	74.63	25.36
Legehare	792	588.8	74.34	73.77	26.33
Addis Ketema	440	330	75.00	68.25	31.75
Sabian	380	228	60.00	68.18	31.82
Kezira	500	320	64.00	68.08	31.92
Dippo	500	350	70.00	60.00	40.00
DDU	270	180	66.67	64.10	35.90
Melka	570	326	57.19	66.15	33.85
Goro	350	210	60.00	78.57	21.43
Total Average	491.8	330.8	67.28	69.48	30.52

**Fig. 2:**
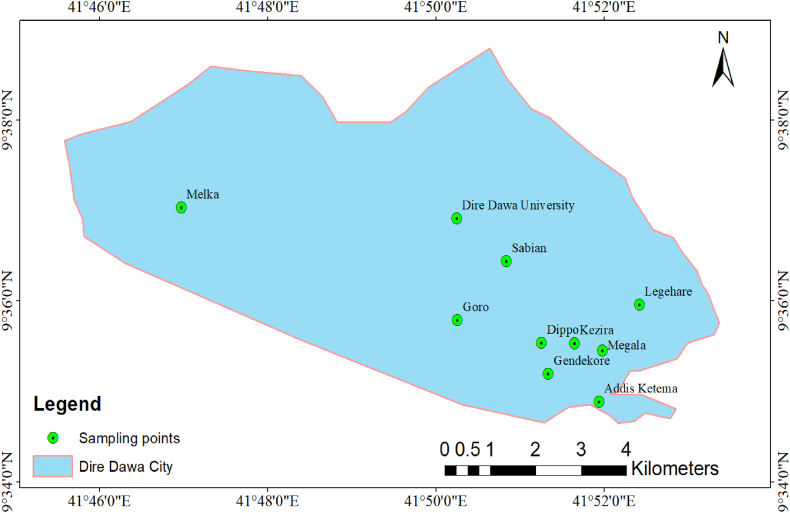
Map showing the geographical location for sampling points of khat waste collected in the Dire Dawa city for data collection.

Furthermore, the specific geographical location of the sampling point for the collection of KW in Dire Dawa City is depicted in Fig. 2.

**Fig. 3:**
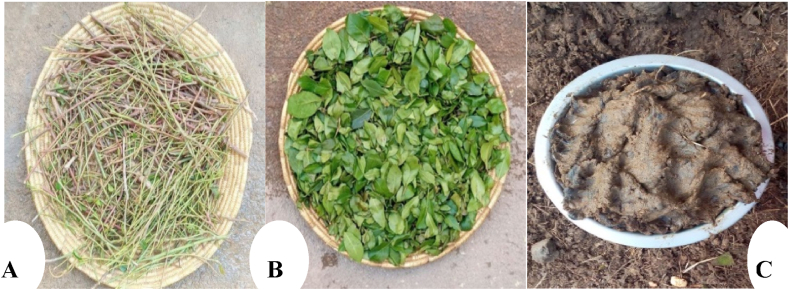
Feedstock (Khat waste steam (A), khat waste leaves (B), and cow dung (C) sample.

### 2.3 Feedstock collection and inoculum

The samples of fresh khat waste (stem and leave) for the study were collected from Dire Dawa city administration (see Fig. 3). The fresh cow dung was collected from the SNNPR Gamo zone Arba Minch town Secha kebele private farms. Furthermore, sheep rumen fluid containing anaerobic inoculum was obtained at a slaughterhouse and used to start up the digesting process. Before seeding, the rumen fluid was kept in a refrigerator at 4 °C. A 10 ml/L active anaerobic inoculum was incubated at 35 ± 3 °C for five days to reduce background gas production and maintain an ideal pH for the process [19,20].

### 2.4 Preparation of substrate

The khat waste samples were collected, cleaned, and chopped manually into 2 mm–4 mm sizes, which were then air-dried for a week. The dried samples were then crushed with a mechanical crusher (model: Solemnly Declare model-350, Turkey) and sieved through a 2 mm sieve size. To prevent undesirable fermentation, both powdered Khat waste and fresh Cow dung were stored at 4 °C before use.

### 2.5 Anaerobic co-digestion

The AD for the generation of methane from khat waste in mono-digestion and co-digestion with cow dung were evaluated in triplicate and grouped into seven treatment proportions. The ratio of the khat waste (KW) and Cow dung (CD) in each reactor was designated as (%KW:%CD) were T-1 (100 % KW), T-2 (50%KW:50%CD), T-3 (33.34 % KW:66.66 % CD), T-4 (25 % KW:75 % CD), T-5 (66.66 % KW:33.34 % CD), T-6 (75 % KW:25 % CD), and T-7 (100 % CD). The mono-digestion and co-digestion mixture proportions were adapted based on the study conducted by Refs. [21,22].

To maintain consistency across all proportions, the organic loading rate of each digester was fixed at 50 g TS per L. According to the study conducted by Ref. [23], the samples should be mixed with water at a ratio of 1:6 of total solids (TS). Accordingly, the contents and the mass of water added along each anaerobic reactor are depicted in Table 2.

**Table 2:** Organic loading rate of lab-scale batch anaerobic treatments combination.

Khat waste (KW)				Cow dung (CD)					
Treatments Designation	Ratio of KW to CD	Avg. KW in (g)	Vol. of water used to mix with 3 % of KOH (6 times TS) of KW in ml	Weight of Fresh CD in (g)	Weight of Dried CD in (g)	CD TS needed water (6 times TS) of CD in ml	Vol. of water in Fresh CD ml	Added water for CD ml	Total Vol. of water mixed with 3 % of KOH (6 times TS) in ml
T - 1	1:0	50	–	247	50	300	197	103	103
T- 2	1:1	25	150	123.9	25	150	98.88	51.12	201.12
T - 3	1:2	16.7	99.96	165.2	33.3	200.04	131.84	68.2	168.16
T - 4	1:3	12.5	75	185.5	37.5	225	148.32	76.68	151.68
T - 5	2:1	33.3	199.92	82.6	16.7	100.08	65.92	34.16	234.08
T - 6	3:1	37.5	225	61.94	12.5	75	49.44	25.56	250.56
T - 7	0:1	–	–	247.8	50	300	197.78	102.22	102.22

The inoculant (sheep rumen fluid) was added to respective digesters for the start-up and robust digestion process. Because [24,25], revealed that the rumen fluid of ruminants was the paramount inoculant to enhance biogas production from lignocellulose biomass, due to the presence of anaerobic bacteria both fungi and methanogens co-cultures.

### 2.6 Pretreatment of khat waste and cow dung

Pretreatment techniques raise the methane content of lignocellulosic biomass by increasing the surface area available for enzymatic hydrolysis, lowering the crystallinity of cellulose, and enhancing the degradation rate [26,27]. Consequently, pretreatment was carried out by mixing 50g of dried milled KW in 100 ml within 3 % of KOH in a conical flask which is closed by rubber cork and kept in an oven at 35 ± 3 °C until an optimum pH was maintained for AD.

### 2.7 Analytical methods

The moisture content (MC), Total Solid content (TS), Volatile Solid content (VS), and Fixed solid content (FS) of feedstock were determined gravimetrically using the hot air oven-drying and ignition method as stated by Ref. [28]. A portable pH meter (model: HQ40d multi, USA) was used to determine the pH which is calibrated using a standard of pH-4 buffer solutions before pH analysis [20].

The determination of total nitrogen content was carried out using the Kjeldahl digestion method as explained by Ref. [29]. Total organic carbon as a percentage basis was estimated using titration methods determined following the procedure outlined by Ref. [30]. The C/N ratio is computed by dividing the carbon by the nitrogen, and the C/N ratio of each combination proportion was calculated using a similar approach based on the anticipated co-digestion ratio.

Elemental analysis of khat waste and cow dung was determined in the EA 1112 Flash CHNS/O- analyzer. Furthermore [31], described a gravimetric method for estimating the amount of hemicellulose in khat waste. Moreover, the lignin and acid detergent fiber (ADF) content on dry weight expressed as a percentage basis in the khat waste was determined gravimetrically using the method defined by the animal nutrition and product quality guidebook [29].

### 2.8 Experimental design and setup

The batch AD experiment was carried out in mesophilic settings at a constant incubated temperature of 35 ± 3 °C utilizing sealed glass bottles with a capacity of 500 mL as batch reactors with a working volume of 400 mL. The experimental setup for a study on batch digester contains a 500 mL conical flask with a plastic stopper for the closing hole of the gas regulator. One plastic tube was taken through the stopper which acts as an outlet for the gas and closed airtight for each batch digester. The system is ensured to be airtight before starting the experiment so that there is no gas leakage or air into the reactor. The detailed experimental setup of the study is depicted in Fig. 5.

**Fig. 4:**
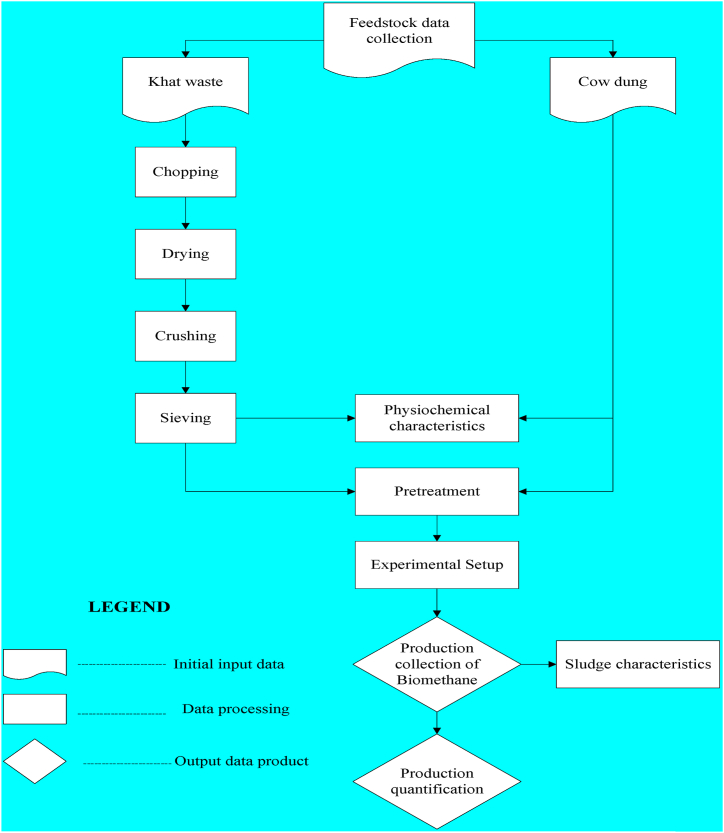
Conceptual frameworks for the experimental study.

All 21 experimental bottles were operated simultaneously and placed inside an oven for better temperature control. The hydraulic retention time (HRT) of the reactor was 27 days and proceeded until low stable gas production was maintained [32]. The general conceptual framework for the experimental study is depicted in Fig. 4.

**Fig. 5:**
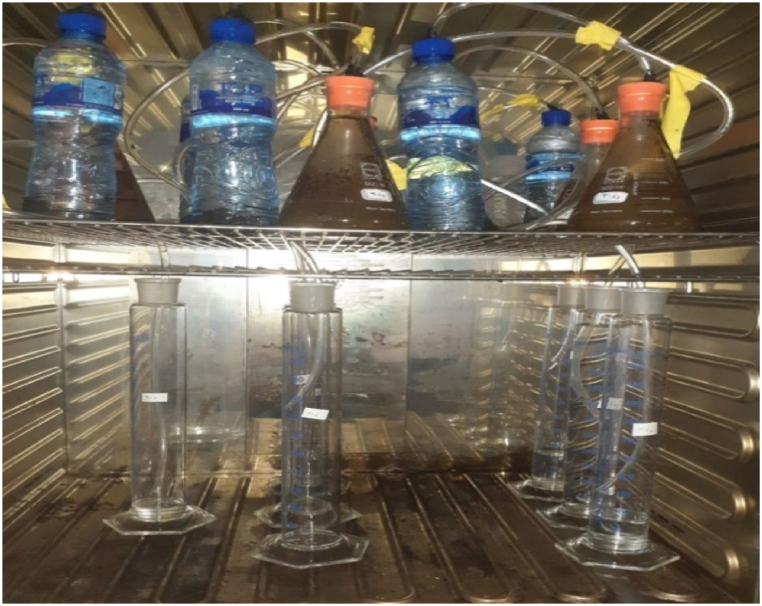
Experimental setup of the study during AD process.

### 2.9 Gas measurement techniques and data analysis

The amount of methane produced by each batch experiment was measured by considering the volume of displaced water. The method consists of allowing the biogas produced to pass through a 3 % KOH solution which is filled in a plastic bottle to capture and convert the CO_2_ fraction present in the biogas into KHCO_3_, and then, to measure volumetrically the fraction of methane produced [33].

The output of the digesters was measured in daily intervals and each treatment was prepared in three replications. Moreover, the methane production potential of the treatments was assessed from the average values of triplicates. In this process, the volume of Bio-Methane Potential in an anaerobic reactor was measured through the adsorption of CO_2_ in an alkaline solution [32].

## 3 Theoretical biomethane potential computation (TBMP)

TBMP based on chemical composition can be obtained by characterizing the element compositions (C, H, N, O, and S) in a substrate [34]. The chemical substrate composition derived by elementary analysis provides the information for stoichiometric calculation of the TBMP depicted in equation (1) using Boyle's formula as stated by Ref. [35]. TBMP of Khat waste and Cow dung was calculated using Equation (1) and Equation (2) according to the study conducted by Ref. [36].

$$C_{a}H_{b}O_{c}N_{d}S_{e}+\left(a-\frac{b}{4}-\frac{c}{2}+\frac{3d}{4}+\frac{e}{2}\right)\;H_{2}O\;\left(\frac{a}{2}+\frac{b}{8}-\frac{c}{4}-\frac{3d}{8}-\frac{e}{4}\right)\;{\text{CH}}_{4}+$$

(1) $$\left(\frac{a}{2}-\frac{b}{8}+\frac{c}{4}+\frac{3d}{8}+\frac{e}{4}\right)\;{\text{CO}}_{2}+{\text{dNH}}_{3}+{\text{eH}}_{2}S$$

TBMP (mLCH_4_gVS^−1^) =

(2) $$\frac{22.4x\left(\frac{a}{2}+\frac{b}{8}-\frac{c}{4}-\frac{3d}{8}-\frac{e}{4}\right)}{12.017a+1.0079b+15.999c+14.0067d+32.065e}$$

Where, a, b, c, d, and e represented the number of moles of C, H, O, N, and S respectively. The elements were calculated by dividing the mass percentage of each element by their molecular weights (g mol^−1^). BMPthAtc in Eq. (2) was expressed in mL CH4gVS^−1^ and the 22.4 factors corresponding to the molar volume (mL mol^−1^) at standard temperature and pressure (STP) [34].

## 4 Result and discussion

### 4.1 Physiochemical characteristics of khat waste (KW) and cow dung (CD)

The Physiochemical characteristics of Khat Waste and Cow dung were characterized before the commencement of the AD process and the average value of triplicate experiments was summarized in Table 3.

**Table 3:** Physicochemical properties of the feedstock.

Parameters (%)	Khat waste (Dry weight basis %)	Cow dung (Dry weight basis %)
TS	26.39 ± 1.19	20.18 ± 1.05
MC	73.61 ± 1.10	79.82 ± 1.05
FS	14.24 ± 0.52	14.64 ± 0.59
VS (as % TS)	85.76 ± 0.84	85.36 ± 0.88
C	38.05 ± 0.72	45.24 ± 0.85
N	1.81 ± 0.05	2.04 ± 0.08
H	4.68 ± 0.09	5.26 ± 0.25
O	55.46 ± 0.85	47.46 ± 1.19
S	ND∗	ND∗
C/N	20.99 ± 0.89	22.22 ± 0.52
Cellulose	33.82 ± 1.01	33.53 ± 0.44
Hemicellulose	22.52 ± 0.45	14.84 ± 0.83
Lignin	15.64 ± 0.37	7.87 ± 0.35

ND∗: Not detected.

As can be seen from Table 3, the total solid content of KW was 26.39 ± 1.19. Out of the total solid, the volatile solid and fixed solid content of the substrate were 85.76 ± 0.84 and 14.24 ± 0.52. This indicates that a large fraction of KW of leave and stem is biodegradable and thus it can serve as an important feedstock for bio-methane production. Correspondingly, the total solid value of khat waste is slightly less than Justicia schimperiana (JS) of (31 %) as reported by Ref. [37] and higher than 15.5 ± 0.3 and 10 % of TS% for Water hyacinth as reported by Refs. [38,39] respectively. Likewise, relatively comparable with 19.06 of TS% of passion fruit peel in a study conducted by Ref. [11]. Moreover, for cow dung, the total solid was 20.18 ± 1.05 which is 0.18 more than the range between 18 and 20 % and comparable with 19.37 ± 0.06 of TS% for cow dung as reported by Ref. [40].

VS% contents of Khat waste and Cow dung samples are as high as 85.76 ± 0.84 % and 85.36 ± 0.88 % respectively. The results infer that a larger percentage of volatile solids implies that the substrate has more organic matter, which produces more biogas [40]. Particularly, the VS% content of Khat waste is comparable with Water hyacinth of 86.2 ± 0.9 % in a study conducted by Ref. [41], and the VS% content of Cow dung is relatively higher than 74.6 ± 1.4 % of VS% content in a study conducted by Ref. [41]. Moreover [42], stated that the high percentage of volatile organic matter designates the availability of flammable organic matter during biomass conversion, Therefore [42], suggested that highly volatile solids are suitable for anaerobic digestion, this showed that both Khat waste and Cow dung are suitable feedstock for bio-methane generation in mono- and co-digestion proportions.

The FS% of Khat waste and Cow dung was found at 14.24 ± 0.52 % and 14.64 ± 0.59 % respectively. Both feedstock amounts of fixed solid were relatively comparable. However, the FS amount of KW and CD is less than 8.5 % of Sunflower reported by Ref. [43]. However, the feedstock with higher fixed solid contents above 20 % is not good for energy conversion [44]. This ascertained that both feedstocks were ideal for biomethane production potential.

It can be observed that the carbon content of the Khat waste and Cow dung were 38.05 ± 0.72 and 45.24 ± 0.85 respectively. However, the carbon content in the Cow dung is higher than in Khat waste and comparable with the 47.00 % of carbon for rice straw as reported by Ref. [45]. The carbon content in the Cow dung is higher than in Khat waste. A higher carbon ratio implies a higher heating value [44].

The nitrogen contents of the Khat waste was 1.81 ± 0.05 which is slightly lower than 2.02 % of Napier grass as reported by Ref. [46], and 2.96 ± 0.34 % of water hyacinth stated by Ref. [47]. Moreover, lower than 2.13 % of the nitrogen content of Justicia schimperiana leaves as stated by Ref. [37]. Likewise, the nitrogen content of cow dung was 2.04 ± 0.08 which is slightly higher than goat manure of 1.8 ± 0.06 % as stated by Ref. [48].

The C/N ratio of both KW and Cow dung for this particular study were 20.99 ± 0.89 and 22.22 ± 0.52 respectively which agreed with the value range of 20:1 to 30:1 for ideal production of biomethane as stated by Ref. [49]. This indicates that KW could serve as a substrate good candidate substrate for biomethane production even without mixing it with cow dung or other animal and human waste provided that it’s available in the area. For the mixture treatments of these substrates, the possible C/N ratio is still around 22:1. Thus, in both substrates, the balance of carbon and nitrogen is good for the bacteria to carry out anaerobic digestion to produce biogas.

The result of the chemical compositional analysis of KW and CW (Table 3), revealed that the KW lignin contents were 15.64 ± 0.37 which is higher than the CD of this particular study but, lower than 20 % of lignin contents for switch grass as stated by Ref. [50] on a dry basis. Correspondingly, the lignin content of CD was 7.87 ± 0.35 which is comparatively lower than 11.6 % of lignin contents for CD as reported by Ref. [51]. The higher lignin amount will hinder the anaerobic degradation of organic matter by anaerobic microorganisms to decompose and convert into biogas. Hence, carrying out pretreatment of KW before AD improves the degradability attribute and higher yield of biogas. Besides, the AcoD process can improve the digestibility of cellulose and hemicelluloses [52].

The Hemicellulose contents of KW and CD were 22.52 ± 0.45 and 14.84 ± 0.83 on a dry basis respectively. The hemicellulose composition of CD was comparable with 15.26 % as reported by Ref. [53]. Likewise, the hemicellulose contents of KW were slightly comparable with the result of [54] reported as 22.15 ± 0.21. Moreover, the cellulose contents of KW were 33.82 ± 1.01 which is relatively lower than Napier grass of 36.81 ± 3.12 % on a dry basis as reported by Ref. [55].

### 4.2 Biomethane production

**Fig. 6:**
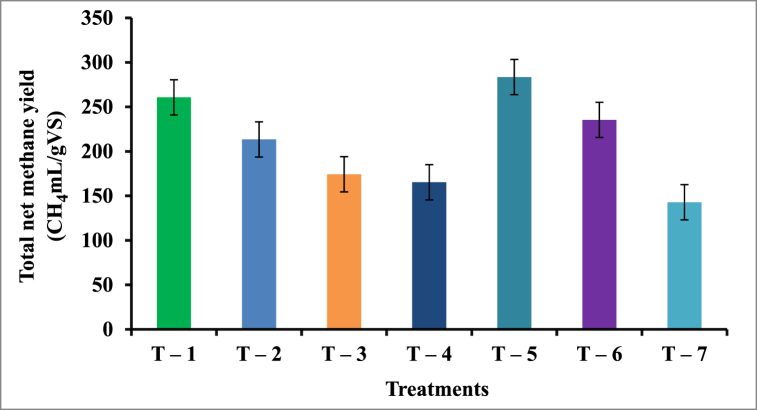
Net methane yield from anaerobic reactors from T-1 to T-7 treatments.

The total net methane yield from the lab-scale batch anaerobic reactors of various treatments is depicted in Fig. 6. The result revealed that the maximum yield of methane was observed in T-5 (2:1) or (66.66 % KW: 33.34 % CD) of 283.52 ± 7.17 CH_4_ mL/g VS followed by T-1 (100 % KW) of 260.69 ± 1.69 CH_4_ mL/g VS, T-6 (75 % KW:25 % CD) of 235.36 ± 8.30 CH_4_ mL/g VS, T-2 (50 % KW: 50 % CD) of 213.42 ± 2.66 CH_4_ mL/g VS, T-3 (33.34 % KW: 66.66 % CD) of 174.24 ± 1.34 CH_4_ mL/g VS, T-4 (25 % KW:75 % CD) of 165.32 ± 5.93 CH_4_ mL/g VS, and T-7 (100 % CD) of 142.83 ± 3.56 CH_4_ mL/g VS.

The result infers that the highest proportion of Khat waste can improve the rate of degradation compared to a sole substrate combination. Congruently, the lower methane production was observed in the T-7 (0:1) ratio of 142.83 ± 3.56mL/gVS, for the sole substrate proportion of cow dung due to the least degradation rate. Hence [56], infer that low methane produced suggests a higher hydrolytic-acidogenic phase than the methanogenic phase in the reactors of the experiments.

The favorable synergetic impact of co-digestion of Khat waste and Cow dung in providing more balanced nutrients and better buffering capacity to maintain stable pH might explain the greater methane output produced from those anaerobic reactors. The digestion of many types of substrates may result in good synergism in the digester [57]. Likewise, the maximum methane yield obtained in this experiment was comparable with 283.55 CH_4_ mL/g VS obtained from co-digestion of water hyacinth and cow manure as reported by Ref. [58].

**Fig. 7:**
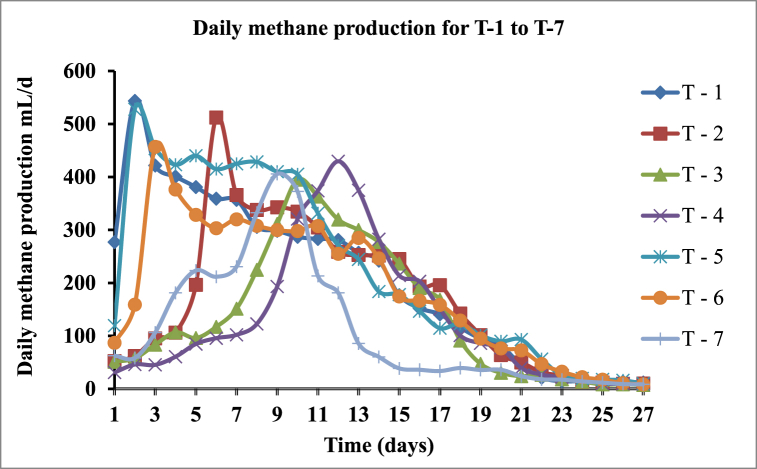
The daily methane production trend for AD system ranging from T-1 to T-7 treatment proportion.

The daily biomethane production potential was measured for about 27 days of the digestion period until gas production was insignificant daily for all seven treatments and the result was depicted in Fig. 7. The daily methane production showed a declining trend with the gradual degradation of degradable organics, and the measured results in each sample were contained by insignificant variations for 24–27 days. It was found that T-5 produced the highest cumulative methane generation of 6069 ± 153.39 ml at 27 days of the digestion period. Comparatively, T-7 produced the lowest of 3048 ± 75.9 ml of cumulative methane production among the seven treatments.

The maximum methane generation in T-5 is due to a well-balanced nutrient quality in the digester, which promotes effective methanogenesis activity. The result showed that co-digestion of Khat waste and cow dung is a potential strategy for producing the highest methane output than sole substrate combination during the AD process. Moreover, the highest methane production of this experiment was higher than that of 5307.8 ml obtained from the co-digestion of fruit and vegetable peal waste mixing with cow dung as stated by Ref. [49].

The khat waste alone T-1 (1:0) ratio has the highest overall daily methane gas output volume as 544 ± 8.14 ml reached after 2 days followed by 512 ± 13.01 ml, 395 ± 13.45 ml, 430 ± 17.78 ml, 528 ± 39.39 ml, 457 ± 30.07 ml, and 406 ± 12.5 ml by the co-digestion of khat waste and cow dung in T-2 (1:1), T-3 (1:2), T-4 (1:3), T-5 (2:1), T-6 (3:1), and cow dung alone T-7 (0:1) proportion at 6 days, 10 days, 12 days, 2 days, 3 days, and 9 days respectively (Fig. 7). The result infers that the overall volume of daily gas production decreases as the proportion of khat waste decreases for the specified digesters. Particularly, the peak methane production appeared in the 2nd to 6th days after the digestion was commenced. Therefore, the faster methane production rate and earlier maximum methane production have resulted in peak production.

More than half of methane production was obtained during 12 days of the digestion period and up to 23 days of the digestion period almost 95 percent of methane gas production was collected. It was observed that methane gas production increased sharply from the first day of digestion and reached a peak value of 544 ± 8.14 CH_4_ mL/d on the 2nd day of the digestion period. However, a lag phase may happen during the first week of the digestion period in which the gas produced might be unusable. Because, in the first three days of the first week, the total gas produced is removed through the drainage of the biogas plant installation, and using the gas produced after this period is currently practiced by the household biogas user in Ethiopia [37].

**Fig. 8:**
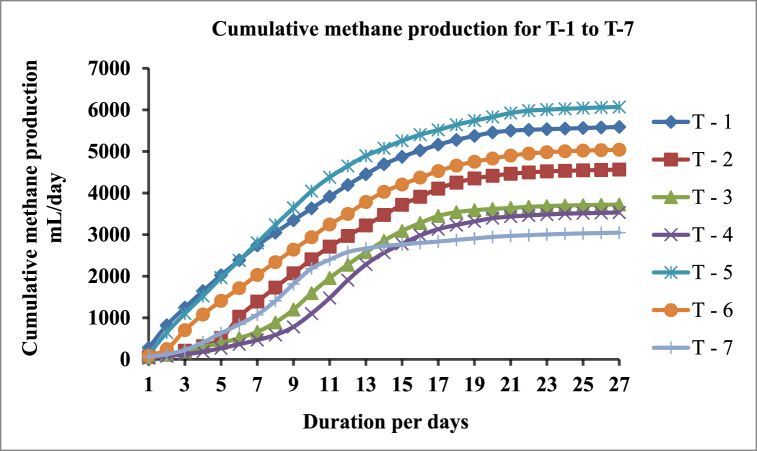
Cumulative methane production trend from T-1 to T-7 treatment proportion from batch anaerobic reactors for 27 days.

The cumulative methane production for seven sample groups during 27 days of the digestion period was depicted in Fig. 8. The result indicates that the highest cumulative biomethane generation was observed in T-5 (2:1) followed by T-6 (3:1), T-1(1:0), T-2 (1:1), T-3 (1:2), T-4 (1:3), and T-7 (0:1) along each ratio of replication from batch anaerobic reactors.

The gradual increase and decrease in the digestion period have a direct influence on the cumulative production of methane for the specified ratio of combination in each reactor. A large amount of gas was produced when a large amount of Khat waste was added. Moreover, the daily methane gas production rate was slightly rapid in the sample group with a large proportion of KW as shown in Fig. 8. As a result, within 27 days of the experiment, a mix ratio of T-5 (2:1) produces more cumulative methane than the sole substrate anaerobic digestion in T-1(1:0) and T-7(0:1) of khat waste and cow dung respectively.

### 4.3 Theoretical biomethane production

The results are unlikely to match the true results because in practice no reaction goes to full completion and we do not have 100 % breakdown of cellulosic materials. Hence, according to the study carried out by Ref. [59], we use a value of f (80 %) to adjust the gas produced under (unrealistic) ideal conditions to the gas produced under real conditions and the results were tabulated in Table 4.

**Table 4:** Results of BMP_th_ and BMP_exp_ in comparison with adjusted BMP values.

Feedstocks	BMP_exp_ (mLCH_4_/gVS)	BMP_th_ (mLCH_4_/gVS)	Adjusted BMP_th_ (mLCH_4_/gVS)
Khat Waste	260.69 ± 1.69	348	278.42
Cow Dung	142.83 ± 3.56	373	298.64

The BMP_th_ of Khat waste is consistent with the garden waste of 336.65 mLCH_4_/gVS and as low as 401.17 mLCH_4_/gVS of vegetable waste as reported by Ref. [34]. However, higher than 221 mLCH_4_/gVS along co-digestion of biological sludge and OFMSW as reported by Ref. [60]. Hence, in the case of determining BMP based on elemental composition, a higher C/N ratio significantly increased methane production [34].

### 4.4 Rate of substrate conversion

#### 4.4.1 TS and VS conversion rate during AD

**Fig. 9:**
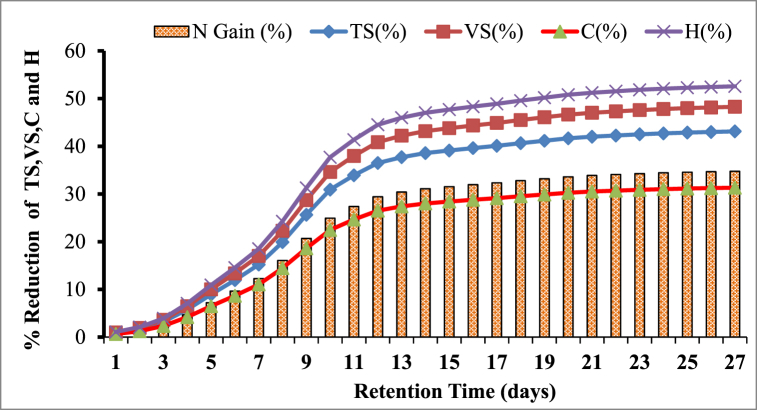
Graph showing the TS and VS conversion rates in cow dung during the digestion period of the AD process.

**Fig. 10:**
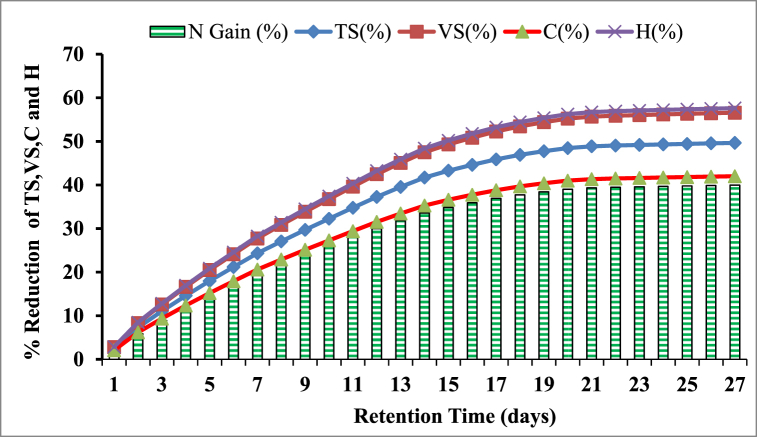
Graph showing the TS and VS conversion rates in Khat waste during the digestion period of the AD process.

The microbial communities in the reactor maximize the use of the substrate for their growth and production, which decreases the key components of the feedstock. As a result, after a certain digestion period, the TS and VS of khat waste and cow dung gradually decreased. The result of the percentage reduction of TS and VS Cow dung and Khat waste was depicted in Fig. 9, Fig. 10 respectively.

The results indicated that in the reactor's TS and VS, removal rates increased steadily over the digestion cycle. For example, khat waste had a TS and VS reduction of 17.98 % and 20.49 % in the first five days, while cow dung had a TS and VS reduction of 8.94 and 10.01 % within the prescribed retention time. This partial improvement in TS and VS elimination may be attributed to comparatively high biomethane generation during the digestion periods of the first week. Since, in the anaerobic digestion process, the amount of dry matter removed is directly proportional to biogas yield [23].

Moreover, more than half of the total TS and VS conversion of khat waste was accomplished in the first 8 days of the digestion period, and more than half of the total TS and VS conversion of cow dung was achieved in the first 9 days of digestion time. As a result, methanogens easily absorbed the soluble organic materials hydrolyzed from the substrate [61].

#### 4.4.2 Elemental conversion during the AD process in khat waste and cow dung

Carbon, nitrogen, hydrogen, and sulfur make up the majority of the organic components of khat waste and cow dung, which serve as the essential nutrients for microbial groups. During the digestion process, the overall biomass, nitrogen, and hydrogen content all decreased significantly, however, the shift in sulfur content was negligible. The result of elemental conversion during anaerobic degradation was tabulated in Table 5.

**Table 5:** Elemental conversion during the AD process in Khat waste and cow dung.

Elemental composition (%)	KW sludge (%)	KW Reduction (%)	Cow dung (CD) (%)	CD sludge (%)	CD Reduction (%)
C	22.07 ± 1.46	42.00	45.24 ± 0.85	31.07 ± 1.34	31.32
H	1.98 ± 0.34	57.62	5.26 ± 0.25	2.50 ± 0.19	52.58
N	2.54 ± 0.72	−39.96	2.04 ± 0.08	2.74 ± 0.44	−34.77
O	73.41 ± 1.07	32.38	47.46 ± 1.19	63.68 ± 0.71	44.71
S	ND∗	ND∗	ND∗	ND∗	ND∗

ND∗-Not detected.

The concentrations of carbon and hydrogen reduction improved as the digestion time proceeded. Moreover, the overall nitrogen content increased steadily until the end of digestion. Specifically, at the end of the digestion period, the maximum carbon and hydrogen elimination from the khat waste reaches 42.00 % and 57.62 %. Likewise, for cow dung, it was about 31.32 % and 52.58 % for carbon and hydrogen respectively. On day 27, the net gain of total nitrogen from KW and CD was 39.96 % and 34.77 % respectively (Table 5). This might be due to the removal of both carbon and hydrogen mostly in the form of biomethane; instead of the escalating in nitrogen content, Moreover, the changes in microbial communities and the residual body of microorganisms, as well as the pretreatment reagent might affect the reduction of nitrogen during AD process. The result infers that the rate of carbon removal was elevated for a long period of digestion time. As a result, the C/N ratio dropped below the optimum level, and the system's biomethane output volume declined drastically.

#### 4.4.3 Lignocellulose degradation rate in the AD process

The rate of Cellulose and Hemicellulose reduction after every digestion period based on the dry weight of sample proportion were depicted in Table 6. The lignin, cellulose, and hemicellulose (LCH) components of khat waste and cow dung are the main carbon sources for anaerobic microorganisms.

**Table 6:** Degradation of lignocellulosic components in khat waste and Cow dung.

Test Days	KW Cum.CH_4_ ml/d	Reduction %		CD Cum.CH_4_ ml/d	Reduction %	
		Cellulose	Hemicellulose		Cellulose	Hemicellulose
1	277	2.20	2.34	63	1.06	0.98
2	821	6.52	6.93	121	2.05	1.89
3	1242	9.87	10.49	227	3.86	3.56
4	1643	13.05	13.88	409	6.93	6.40
5	2024	16.08	17.09	632	10.72	9.89
6	2383	18.93	20.12	844	14.31	13.20
7	2740	21.76	23.14	1074	18.22	16.81
8	3045	24.19	25.72	1407	23.87	22.02
9	3344	26.56	28.24	1813	30.75	28.37
10	3631	28.84	30.66	2185	37.07	34.20
11	3914	31.09	33.05	2399	40.69	37.54
12	4195	33.32	35.42	2580	43.76	40.38
13	4452	35.36	37.60	2666	45.21	41.72
14	4694	37.29	39.64	2726	46.24	42.66
15	4870	38.69	41.13	2765	46.89	43.27
16	5023	39.90	42.42	2801	47.50	43.83
17	5163	41.02	43.61	2834	48.08	44.36
18	5279	41.94	44.58	2874	48.74	44.97
19	5374	42.69	45.38	2909	49.35	45.53
20	5455	43.34	46.07	2945	49.96	46.09
21	5500	43.69	46.45	2969	50.37	46.47
22	5521	43.86	46.63	2987	50.67	46.75
23	5535	43.97	46.75	3005	50.96	47.02
24	5550	44.09	46.87	3019	51.20	47.24
25	5563	44.20	46.98	3030	51.40	47.42
26	5577	44.30	47.10	3039	51.55	47.56
27	5589	44.40	47.20	3048	51.70	47.70

The rate of lignocellulose degradation can be used to assess the effectiveness of the pretreatment and the anaerobic digestion efficiency [62]. However, the recalcitrant lignin network prevents cellulose crystallinity and hydrolysis. The high contents of VS, cellulose, hemicellulose, and glucose in khat waste and cow dung infers the availability content of organic matter.

The results of the experiments showed that the key components of the KOH (Potassium Hydroxide) pretreated sample differed significantly from that of the sole substrate of the sample of KW and CD, in maximal lignin network degradation, successful reduction of cellulose, and hemicellulose structure complexity, and a strong substrate for anaerobic microbial attack. This could make the anaerobic digestion process easier by increasing microbial accessibility.

The lignocellulose degradation of Khat waste was over 44.4 % of cellulose and 47.2 % of hemicellulose was converted to methane gas during 27 days of digestion period. Correspondingly, 51.7 % of cellulose and 47.7 % of hemicellulose of cow dung were converted to methane gas during the prescribed range of digestion periods.

## 5 Conclusion

The C/N ratio of KW and CD was 20.99 and 22.22 respectively which is found under the prescribed range for optimal biomethane production of 20–30. This showed that khat waste biomass was highly organic and contained less nitrogen. Therefore, both substrates can be candidate feedstock for methane production in both mono and co-digestion proportions.

From the batch experimental study, the biomethane production potential in all digesters ranged from 283.52 ± 7.17 CH_4_ mL/g VS to 142.83 ± 3.56 CH_4_ mL/g VS. Moreover, the peak cumulative daily biomethane production was produced in a mixture of KW to CD at the ratio of 2:1 (T-5) which generated 6069 ± 153.39 ml throughout all digestion periods under mesophilic conditions. The result infers that the co-digestion proportion could produce the maximum biomethane than the sole substrate anaerobic digestion.

Therefore applying co-digestion with appropriate co-substrate material is supreme to improve the digestion process. The Khat waste was finally assessed for its suitability for biomethane production use. Even though lower methane production was found in the sole substrate proportion of cow dung, proven as good methane potential and it is possible to apply to supplement their scarce energy option.

## CRediT authorship contribution statement

**Henok Akililu Legesse:** Conceptualization. **Wagene Hailu Debele:** Writing – review & editing, Writing – original draft, Validation, Supervision, Resources, Project administration, Methodology, Investigation, Funding acquisition, Formal analysis, Data curation, Conceptualization. **Akiber Chufo Wachemo:** Conceptualization.

## Data code availability statement

The data that has been used is confidential.

## Declaration of competing interest

The authors declare the following financial interests/personal relationships which may be considered as potential competing interests:Wagene Hailu reports statistical analysis, travel, and writing assistance were provided by Adigrat University College of Engineering and Technology. Wagene Hailu reports a relationship with Adigrat University College of Engineering and Technology that includes: employment. Wagene Hailu has patent pending to pending. Henok Akililu Legesse, Akiber Chufo Wachemo, If there are other authors, they declare that they have no known competing financial interests or personal relationships that could have appeared to influence the work reported in this paper.
